# Gripping Success Metric for Robotic Fruit Harvesting

**DOI:** 10.3390/s25010181

**Published:** 2024-12-31

**Authors:** Dasom Seo, Il-Seok Oh

**Affiliations:** 1 Department of Computer Science & Artificial Intelligence, Jeonbuk National University, Jeonju-si 54896, Republic of Korea; dasomseo@jbnu.ac.kr; 2 Center for Advanced Image and Information Technology (CAIIT), Jeonbuk National University, Jeonju-si 54896, Republic of Korea

**Keywords:** computer vision, robot harvesting, robotic grasping, grasp detection, object detection

## Abstract

Recently, computer vision methods have been widely applied to agricultural tasks, such as robotic harvesting. In particular, fruit harvesting robots often rely on object detection or segmentation to identify and localize target fruits. During the model selection process for object detection, the average precision (AP) score typically provides the de facto standard. However, AP is not intuitive for determining which model is most efficient for robotic harvesting. It is based on the intersection-over-union (IoU) of bounding boxes, which reflects only regional overlap. IoU alone cannot reliably predict the success of robotic gripping, as identical IoU scores may yield different results depending on the overlapping shape of the boxes. In this paper, we propose a novel evaluation metric for robotic harvesting. To assess gripping success, our metric uses the center coordinates of bounding boxes and a margin hyperparameter that accounts for the gripper’s specifications. We conducted evaluation about popular object detection models on peach and apple datasets. The experimental results showed that the proposed gripping success metric is much more intuitive and helpful in interpreting the performance data.

## 1. Introduction

With the acceleration of agricultural automation due to labor shortages and efficiency requirements, various agricultural robots have been developed, among which research on harvesting robots for orchards and greenhouses has been particularly active [1,2,3,4,5,6,7,8,9]. However, challenges remain in adapting these technologies to the real world, especially in grasp detection for complex agricultural environments. Robotic harvesting studies typically follow two stages: first, identifying and locating the target fruits; and second, cutting or picking the fruits and loading them into a storage system [8].

One of the important issues in developing a harvesting robot system is to evaluate the gripping success rate for the fruit detection models. The evaluation data will help farmers predict how much the labor cost will be saved. Additionally, the robot engineer can use the data to optimize the robot settings, like setting up the opening margin of the gripper. The existing research on robot harvesting uses the de facto standard metric of average precision (AP). The AP score is a metric based on the intersection-over-union (IoU), which measures the overlap between predicted and ground-truth bounding boxes [10]. However, the AP has limitations in predicting the success of robotic picking because predicted boxes with the same IoU may succeed or fail in picking depending on the overlapping shape.

To address this gap, we propose a new metric called gripping success, designed to better reflect robotic picking performance by directly deciding the success of a picking trial. The gripping success generates a “grip” from the bounding box predicted by the object detector and determines success or failure based on whether the ground truth bounding box falls within the range of the grip. The grip can be generated in various forms depending on the α parameter, which considers the opening margin of the gripper. By adjusting this margin, the gripping performance in different scenarios can be evaluated. The gripping success is ultimately represented by precision, recall, and F1-score. Precision and recall, respectively, provide insights into the efficiency of the robot’s picking trials and the operational efficiency of the robot from the orchard’s perspective.

Modern robotics is actively exploring end-to-end grasp detection that predicts the grip pose directly from the input image, rather than undergoing two stages of object detection and grasp planning [11,12,13]. This grasp detection scheme is also applied to agricultural robotics studies in controlled environments where the target fruits are placed on a white background and the datasets used are not publicly available [14]. The gripping success metric proposed in this paper can also be applied to this end-to-end scheme, since the proposed metric is not dependent on the processing stages.

The AP metric and the gripping success metric are measured by the experiments performed with two datasets acquired from actual apple and peach orchards. Three deep learning-based detection models, Faster R-CNN [15], YOLO-v8 [16], and Swin Transformer [17], were fine-tuned to these datasets. The performance data are interpreted from the perspective of benefits that will be given to the expert people involved in the harvesting work. The conclusion is that, while the traditional AP metric is very poor in helping the experts, the information in gripping success data is much richer and helps them in various manners. The remainder of this paper is structured as follows: Section 2 introduces the AP score, a conventional object detection metric, along with the proposed metric, gripping success, and its rationales for robotic harvesting. Section 3 presents the experimental results, comparing COCO AP and gripping success on the apple and peach image datasets. Section 4 provides a detailed discussion of the gripping success results, and Section 5 concludes this paper with a summary of future research directions.

## 2. Materials and Methods

In this section, we describe conventional metric based on IoU and propose the “gripping success metric” for robotic harvesting as a novel evaluation. We assume that the robot uses a two-finger-type gripper.

### 2.1. Conventional Metric Based on IoU

**Figure 1:**
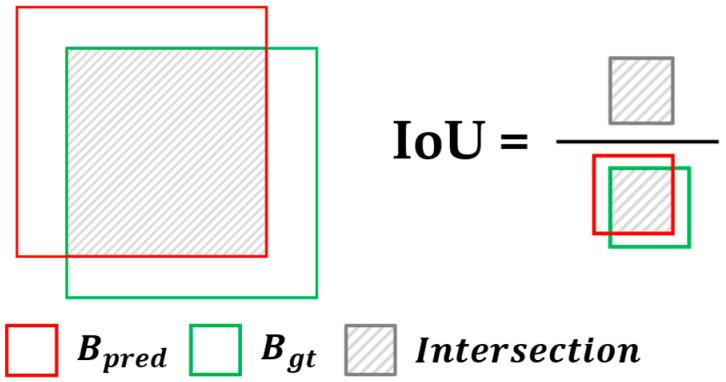
Scheme of intersection over union (IoU).

In general cases, the object detection approach is utilized to develop harvesting robots [1,7,8]. Researchers have required selecting the models with an average precision (AP) score, a common metric to measure the performance of object detection [10]. The AP metric is computed through complicated processes and concepts; however, the AP metric is basically based on intersection-over-union (IoU) to classify each predicted result as true or false. IoU is scored as an overlapping area divided by a union area between the predicted bounding box $B_{pred}$ and the ground truth bounding box $B_{gt}$, described in Equation (1) and Figure 1. Measuring IoU results in T/F outcomes within specific thresholds. The prediction with higher IoU than threshold is considered a true positive (TP). Conversely, if the IoU falls below the threshold, the prediction is classified as a false positive (FP). Miss detections, not predictions for a ground truth, are considered false negatives (FN).

The AP metric relies on evaluating the model’s precision and recall, where true–false results are determined based on the IoU. Precision represents the proportion of correct detections of all predicted objects, while recall represents the proportion of correct detections of all ground truth objects. These precision and recall calculations are described in Equation (2). The AP is a method of evaluating performance by plotting a precision–recall curve (P-R curve) based on precision and recall calculated at various thresholds, then calculating the area under the curve (AUC). Among the two available metrics of Pascal VOC and COCO, we use the COCO metric.

(1) $$IoU=\frac{B_{gt}\cap B_{pred}}{B_{gt}\cup B_{pred}},$$

(2) $$Precision=\frac{TP}{TP+FP}\;Recall=\frac{TP}{TP+FN},$$

### 2.2. Gripping Success Metric

#### 2.2.1. Basic Design for Two-Parallel Grippers

**Figure 2:**
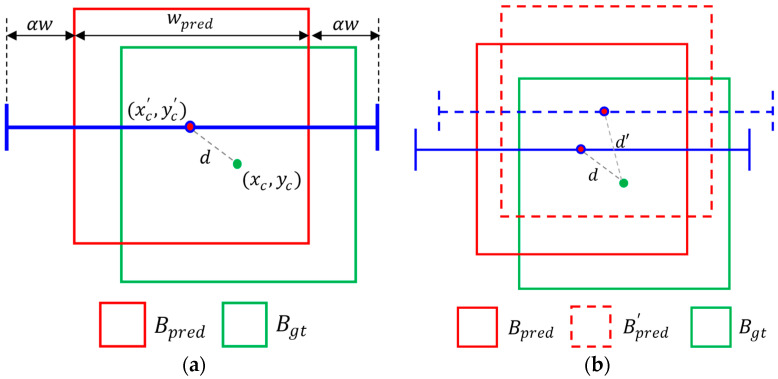
The schemes of a grip. (**a**) The green box is a ground-truth bounding box $B_{gt}$, and the red box is a predicted bounding box $B_{pred}$. The grip is marked by a blue line. The grip consists of the center point of $B_{pred}$ and lines that represent opening of the gripper. The gripper is assumed to open with a specific margin αw, equal to the α factor of the predicted box width $w_{pred}$ of $B_{pred}$. (**b**) shows the case in which duplicated prediction occurred. The duplication bounding box $B_{pred}^{\prime }$ and its grip are described in dashed lines. To remove duplication, the metric compares the distance between the center points, *d* and *d’*.

Figure 2a is the scheme explaining the gripping success metric. The grip marked with thick blue lines is placed over the prediction box with the margin αw. When the fruit width is w, the margin αw is given at both sides to allow the robot gripper to grasp the fruit safely. If the ground truth (GT) box is enclosed entirely within the grip horizontally, the grip is considered a success. Otherwise, the grip is considered a failure. The grip in Figure 2a is a successful instance. When we reduce the margin by half, the grip will be a failure, since the right finger will be positioned outside the GT box.

Figure 2b illustrates a complex situation where multiple predictions are generated for a GT box. Although the detection module applies the non-maximum suppression to the initial detection result to remove the overlapping predictions, the overlapping cases remain. To resolve the cases, we apply the Hungarian algorithm. Algorithm 1 shows the overall procedure of our calculation of the gripping precision and recall. In the inputs, N and M are the number of GT and prediction boxes, respectively. The algorithm uses two tables, S and D, which store the success/fail information and distance between the centers of the GT and prediction boxes, respectively. The rows and columns of the table correspond to the GT and prediction boxes, respectively. Therefore, the size of the tables is N×M.

The double **for** statements of Algorithm 1 fill the tables S and D. The function *Hungarian* applies the Hungarian algorithm, which selects the most relevant prediction box for each GT box, considering whole pairs globally. For the details of the Hungarian algorithm, we refer the readers to [18]. The result returned by the function *Hungarian* is the table $S^{\prime }$, in which a row is a binary vector containing one or zero elements with value 1. The row with zero elements with value 1 becomes the false positive. The row with one element with value 1 becomes the true positive. The function *sum*($S^{\prime }$) counts the number of true positives. Therefore the $sum(S^{\prime })/M$ and $sum(S^{\prime })/N$ calculates the gripping precision and recall, respectively.
**Algorithm 1:** Gripping success**Input:**$\;\mathrm{ground}\;\mathrm{truth}\;\mathrm{boxes}\left\{X,\;Y,\;W,\;H\right\}\in R^{N\times 4}$ $\;\mathrm{predicted}\;\mathrm{boxes}\;\left\{X^{\prime },\;Y^{\prime },\;W^{\prime },\;H^{\prime }\right\}\in R^{M\times 4}$    margin α **Output:** picking success precision *P***,** picking success recall *R*1:**for**i←1toNdo2:
$l_{gt}\leftarrow x_{i}-w_{i}/2$3:
$r_{gt}\leftarrow x_{i}+w_{i}/2$4:
$diag=\sqrt{w_{i}^{2}+h_{i}^{2}}$5:
**for**j←1 to M do6:

$l_{grip}\leftarrow x_{j}^{\prime }-w_{j}^{\prime }/2-\alpha w$7:

$r_{grip}\leftarrow x_{j}^{\prime }+w_{j}^{\prime }/2+\alpha w$8:

**if** $l_{grip}<l_{gt}$ **and** $r_{grip}>r_{gt}$ **then**9:


$S\left[i,j\right]\leftarrow 1$ // s*uccess*10:

**else**11:


$S\left[i,j\right]\leftarrow 0$ // failure 12:

**end if**13:

$d_{ij}=\sqrt{{\left(x_{i}^{\prime }-x_{j}\right)}^{2}+{\left(y_{i}^{\prime }-y_{j}\right)}^{2}}$14:

$D\left[i,j\right]\leftarrow d_{ij}/diag$ // c*enter distance ratio*15:
**end for**16:**end for**17:// *Remove duplication*18:$S\prime \leftarrow Hungarian\left(S,D\right)$19:$P=sum(S^{\prime })/M$20:$R=sum(S^{\prime })/N$21:**return**  
*P, R*


#### 2.2.2. Expansion to Soft Grippers

The initial designs of grip scheme and gripping success are focused on the basic two-parallel gripper type. However, with the introduction of soft robotics technologies, modern harvesting robots use a variety of gripper types, such as 3- or 4-finger grippers, tendon-driven grippers, and suction-type grippers [19,20,21,22]. To enhance the applicability of gripping success to modern grippers, an expanded version of the metric is presented. Note that the target fruits are assumed to be exclusively round shaped, such as apples, peaches, tomatoes, and citrus fruits.

**Figure 3:**
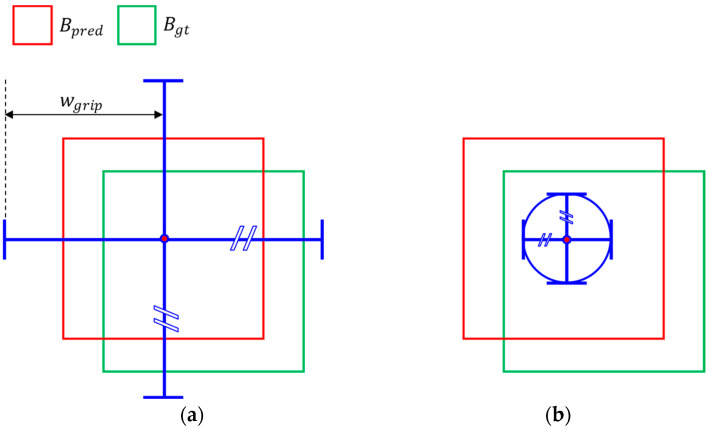
The expanded grip schemes. Each scheme is corresponding to (**a**) 4-finger type grippers and (**b**) suction-type grippers. The grips are marked by blue lines and circle. The red circles are the center points of predicted boxes.

Figure 3 shows the expanded grip scheme for (a) 4-finger type grippers and (b) suction-type grippers. In Figure 3a, the gripper’s width in all directions is the same, denoted as $w_{grip}$, and the suction-type grip scheme is represented as a circle with a radius of $w_{grip}$, as shown in Figure 3b. The value of $w_{grip}$ is defined by Equation (3).

(3) $$w_{grip}=\left\{\begin{array}{c} \left(1+\alpha \right)\times \frac{\bar{w+h}}{2},\;4-finger\\ \left(1-\alpha \right)\times \frac{\bar{w+h}}{2},\;suction-type \end{array}\right.$$

Assuming the target is a sphere with a radius equal to half the average of the width *w* and height *h* of the prediction box, the margin of the gripper is determined by multiplying the radius by α coefficient. For a 4-finger type gripper, which needs to open wider than the target, the margin is added to the baseline, while for a suction-type gripper, which has a smaller area than the target, the margin is subtracted. When the gripper can fully enclose the target, it will always succeed in grasping regardless of the approach direction if the target is spherical. Therefore, the orientation of the gripper is not considered.

Line 2–3 and line 6–8 of Algorithm 1 should also be modified. Algorithms 2 and 3 illustrate expanded versions for Figure 3a and Figure 3b, respectively. These algorithms focus on the calculation of grips and gripping success only. GT, grip, and success criteria for the top and bottom side have been added. Algorithm 2 is a simple extension of Algorithm 1 for the top and bottom side, whereas Algorithm 3 adjusts for the suction gripper by subtracting the margin and reversing the success criteria. For the suction gripper, it is considered successful if the grip is fully contained within the ground truth.
**Algorithm 2:** Gripping success for 4-finger grippers**Input:**$\;\mathrm{ground}\;\mathrm{truth}\;\mathrm{boxes}\left\{X,\;Y,\;W,\;H\right\}\in R^{N\times 4}$ $\;\mathrm{predicted}\;\mathrm{boxes}\;\left\{X^{\prime },\;Y^{\prime },\;W^{\prime },\;H^{\prime }\right\}\in R^{M\times 4}$    margin α **Output:** success matrix **S**1:**for**i←1toNdo2:
$l_{gt}\leftarrow x_{i}-w_{i}/2$3:
$r_{gt}\leftarrow x_{i}+w_{i}/2$4:
$t_{gt}\leftarrow y_{i}-h_{i}/2$5:
$b_{gt}\leftarrow y_{i}+h_{i}/2$6:
**for** 
j←1 to Mdo
7:

$w_{grip}=(1+\alpha )\times (w_{j}^{\prime }+h_{j}^{\prime })/4$8:

$l_{grip}\leftarrow x_{j}^{\prime }-w_{grip}$9:

$r_{grip}\leftarrow x_{j}^{\prime }+w_{grip}$10:

$t_{grip}\leftarrow y_{j}^{\prime }-w_{grip}$11:

$b_{grip}\leftarrow y_{j}^{\prime }+w_{grip}$12:

$\mathrm{if}l_{grip}<l_{gt}$ **and** $r_{grip}>r_{gt}$ **and** $t_{grip}<t_{gt}$ **and** $b_{grip}>b_{gt}$ **then**13:


$S\left[i,j\right]\leftarrow 1$ // success
14:

**else**
15:


$S\left[i,j\right]\leftarrow 0$ // failure16:

**end if**17:
**end for**18:**end for**19:**return S**
**Algorithm 3:** Gripping success for suction-type grippers**Input:**$\;\mathrm{ground}\;\mathrm{truth}\;\mathrm{boxes}\left\{X,\;Y,\;W,\;H\right\}\in R^{N\times 4}$ $\;\mathrm{predicted}\;\mathrm{boxes}\;\left\{X^{\prime },\;Y^{\prime },\;W^{\prime },\;H^{\prime }\right\}\in R^{M\times 4}$    margin α **Output:** success matrix **S**1:fori←1toNdo2:
$l_{gt}\leftarrow x_{i}-w_{i}/2$3:
$r_{gt}\leftarrow x_{i}+w_{i}/2$4:
$t_{gt}\leftarrow y_{i}-h_{i}/2$5:
$b_{gt}\leftarrow y_{i}+h_{i}/2$6:
forj←1 to M d7:

$w_{grip}=(1-\alpha )\times (w_{j}^{\prime }+h_{j}^{\prime })/4$8:

$l_{grip}\leftarrow x_{j}^{\prime }-w_{grip}$9:

$r_{grip}\leftarrow x_{j}^{\prime }+w_{grip}$10:

$t_{grip}\leftarrow y_{j}^{\prime }-w_{grip}$11:

$b_{grip}\leftarrow y_{j}^{\prime }+w_{grip}$12:

$\mathrm{if}l_{grip}>l_{gt}$ **and** $r_{grip}<r_{gt}$ **and** $t_{grip}>t_{gt}$ **and** $b_{grip}<b_{gt}$ **then**13:


$S\left[i,j\right]\leftarrow 1$ // success14:

**else**
15:


$S\left[i,j\right]\leftarrow 0$ // failure16:

**end if**17:
**end for**18:**end for**19:**return S**

### 2.3. Rationales of Gripping Success Rate as a Good Metric for Robot Grasping

**Figure 4:**
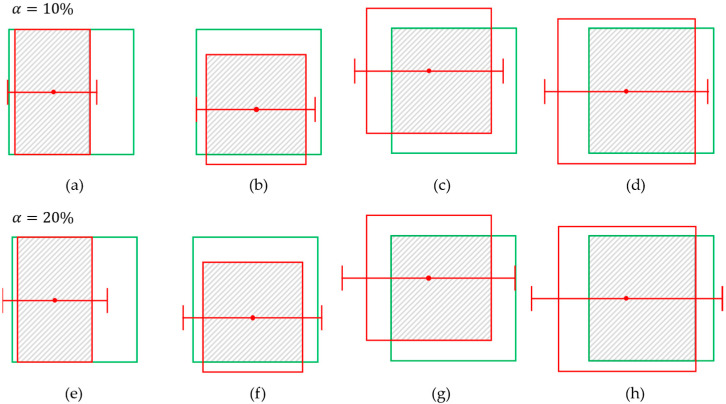
Various predicted bounding boxes at IoU = 0.6 and grasp results for a=10% (**a**–**d**) and a=20% (**e**–**h**). The red boxes represent the predicted boxes, while the green boxes represent the ground truths.

**Figure 5:**
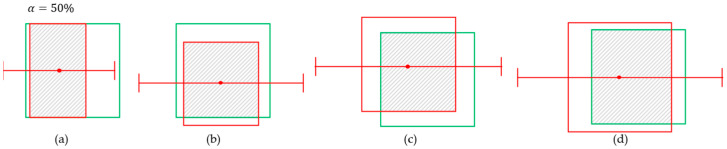
The grasp results for a=50%. (**a**) fails to grasp though the gripper is opened double of the width. (**b**–**d**) succeed while they fail for a=10%. The red boxes represent the predicted boxes, while the green boxes represent the ground truths.

Determining true or false predictions based on IoU score is reasonable when the purpose is simply to evaluate the object detection, focusing on their identity and location. In the case of robotic grasping, however, how the targets are localized in terms of four sides precisely becomes a more crucial point. Figure 4 and Figure 5 show four cases of prediction with 0.6 IoU and their grips. The grips are generated where α is set to 10% (a–d), 20% (e–h), and 50% (Figure 5). In most object detection cases, an IoU threshold of 0.5 or 0.6 is commonly used, and the predictions shown in Figure 4 and Figure 5 would all be classified as true in the context of object detection. However, when examining the generated grips, it is observed that in the case where α is set to 10%, gripping fails in all cases. In the case where α is set to 20%, (f) and (h) are successful, while (e) and (g) fail. Figure 5a fails again even though α=50%. These results demonstrate the reason why the IoU criterion is unreasonable and has limitations in the context of robotic harvesting. Though the detector defines the predicted bounding box as true, the situation can occur where the robot fails to pick the fruit in real. Even though the IoU scores are the same, the predictions can obtain different success or failure in robot picking. These results can cause confusion when the object detection metric is applied to robot harvesting.

The gripping success increases as the margin α increases in general since a larger opening of gripper is more probable to enclose a target object. The gripping success metric represents strict when α is close to 0, indicating the minimal disturbance with neighboring objects. Note that a large α is more probable that the success declaration by our gripping success metric will finally fail due to neighborhood fruits or objects like branches. In this paper, we measure gripping success with α in range 0.05 to 0.5 in Section 3, representing strict score to coarse score. This research does not consider α over 0.5 since the gripper opening is double of bounding box when α is 0.5. The analysis with varying α will be helpful in deciding the grasp planning of harvesting robots.

**Figure 6:**
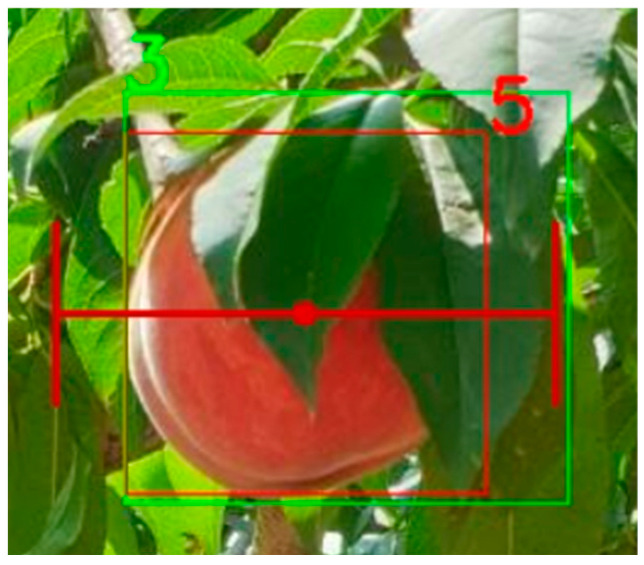
The example image of gripping success. Green boxes are ground truth and red boxes are predictions. The grip is generated with α=20%.

Figure 6 shows actual example image applying gripping success with α=0.2 The green box is GT bounding box and red one is predicted bounding box. The IoU is 0.7. Though the IoU is good enough for object detection, the grip does not include the GT and results in picking failure. Assuming the size of the peach is 10 cm, even with the gripper opening 2 cm on each side to provide a margin, it still fails to grasp the peach. This suggests that evaluating the detection models for robotic picking based solely on IoU is not reasonable.

## 3. Results

In this section, we present the experimental results for gripping success and compare them with the conventional evaluation metric, the MS-COCO metric (hereafter referred to as COCO metric or COCO AP). Since COCO AP is widely used in the field of object detection and evaluates a broader range of conditions, it has been selected for comparison. The experiments were conducted on the most popular object detection models used in robotic harvesting: Faster R-CNN [5,23,24,25], YOLO-based models [23,24,25], and Swin Transformer [26,27]. These models were trained using the apple [28] and peach image datasets [26] and evaluated based on both COCO AP and gripping success.

### 3.1. Datasets

To simulate robot harvesting similar to a real scene, we experimented using the fruit image datasets captured in complex orchard environments. We also considered the bounding box labels, including the obscured fruits, since it is more suitable for evaluating gripping success or failure. In this paper, image datasets for two kinds of fruit, an apple [28] and a peach [26], are used. The apple image dataset, NIHHS-JBNU, consists of 199 images captured in an apple (*Malus pumila Mill*, Hongro) orchard. There are a total of 13,260 labels in the dataset, with an average of 67 labels per image. The peach image dataset consists of 125 images of a peach (*Prunus persica* (L.) Batsch, Mihong), with 1077 labels in total. The images in the apple dataset include many apples per image because they were taken to capture the entire view of the tree, and the apples are relatively very small in size. Conversely, the peach image dataset was captured from various angles and viewpoints, and the sizes of the peaches vary as well.

All the detection models were trained using each of these two datasets. For the apple dataset, we divided the data into an 8:2 ratio, with 160 images for training and 39 images for validation. For the peach dataset, we followed the provided split, using 99 images for training, 13 images for validation, and 13 images for testing. Finally, the validation split of the apple dataset and the test split of the peach dataset were used to evaluate COCO AP and gripping success. The detailed information of the dataset is listed in Table 1.

### 3.2. Model Training

In this paper, three popular object detection models—Faster R-CNN, YOLO v8, and Swin Transformer—were trained using the apple and peach image datasets mentioned in Section 3.1. All the models were trained using transfer learning, i.e., fine-tuning the pre-trained weights with each of the two datasets. When parameter settings differ depending on the dataset, the parameters for each model are described in the format of apple/peach in the following sentences. Faster R-CNN was trained with a learning rate of 0.001 using an SGD optimizer for 32 epochs, with the same settings applied to both the apple and peach datasets. Specifically for the apple dataset, CIoU loss [29] was used instead of L1 loss [15]. YOLO v8 was trained with a learning rate of 0.00172/0.00261 using an AdamW optimizer for 300 epochs with early stopping enabled. For the Swin Transformer, the learning rate and optimizer are set to 0.0001 and AdamW, respectively. The COCO AP performances of the trained models are presented in Table 2 and Table 3 for apple and peach, respectively.

### 3.3. Comparisons of Evalutation Metrics

**Figure 7:**
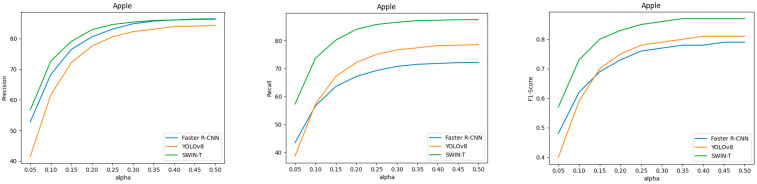
Gripping success precision, recall, and F1-score curves for the apple dataset. All the curves are plotted according to the value of α.

**Figure 8:**
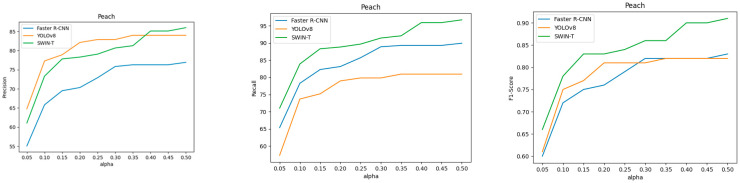
Gripping success precision, recall, and F1-score curves for the peach dataset. All the curves are plotted according to the value of α.

In this section, we compare COCO AP and gripping success for the three popular object detection models discussed in Section 3.2. Table 2 and Table 3 provide a direct comparison of COCO AP and gripping success scores on the apple and peach datasets, respectively. COCO AP is measured at different IoU thresholds: AP50, AP75, and AP50-95. Gripping success is evaluated at various α values, ranging from 0.05 to 0.50. Figure 7 and Figure 8 show the precision, recall, and F1-score curves of gripping success for each dataset. All the curves are plotted according to the value of α. In addition, we note that detection results with a confidence score of 0.5 or higher were used to calculate gripping success, and the NMS threshold of the object detector was set to IoU ≥ 0.7.

For COCO AP in Table 2, the Swin Transformer consistently outperforms the other models. Swin Transformer achieves an AP50 of 86.3, an AP75 of 56.7, and the best AP50-95 score of 52.3, making it the top performer across all IoU thresholds. In comparison, YOLO v8 also shows strong performance but falls behind the Swin Transformer, especially in the AP75 and AP50-95. Faster R-CNN performs the worst compared to the other models.

The gripping success metrics, including precision, recall, and F1-score, are relevant for estimating the picking robot’s ability. As the α-value increases, all models show an improvement in precision, recall, and F1-score, indicating that larger margins for the gripper enhance the gripping success. Gripping success recall presents how many fruits can be picked out of the total, which is crucial for robotic harvesting, as high recall indicates that the robot can effectively perform the task without missing the fruits. Precision, on the other hand, reflects how many predicted fruits are correctly picked. As the gripping success precision increases, it indicates fewer false predictions and more precise localization, reducing cases where fruits cannot be picked. High precision even at small α values suggests that the model can perform accurate picking by minimizing the possibility of touching the nearby fruits or branches, with bounding boxes closely matching the ground truth. The F1-score, being the harmonic mean of precision and recall, shows how balanced and reliable the model is across both precision and recall. A higher F1-score indicates strong and consistent performance in both precision and recall.

Specifically, for example, Swin Transformer shows a precision of 56.7 and recall of 57.31 at α = 0.05 in Table 2. This means that when the size of apple is 10 cm and a robot gripper opening 11 cm approaches the apple, about 56 among 100 picking trials are expected to safely grasp the apples. Increasing α to 0.15 amounts to the gripper opening 13 cm. In this case, the precision is 79.1. About 79 among 100 picking trials are expected to safely grasp the apples. However, as the gripper opening becomes larger, other negative factors, like damaging other apples and/or touching the branches, become stronger.

YOLOv8 demonstrates a similar trend, with precision reaching 84.30, recall increasing to 78.60, and F1-score peaking at 0.81 at α = 0.50. Swin Transformer excels in gripping success, achieving the highest precision of 80.59, recall of 81.71, and F1-score of 0.81 at α = 0.50, highlighting its superior ability to both detect and pick objects compared to the other models.

Figure 7 presents the gripping success precision, recall, and F1-score curves, derived from the values in Table 2. In Figure 7, Swin Transformer outperforms all the metrics regardless of α value, especially recall. Swin Transformer also shows convergence in performance starting from α value above 0.3. This convergence suggests that if the gripper’s opening is at least 30% of the fruit’s size, robotic picking using Swin Transformer can maintain stable performance in any situation.

Overall, COCO AP is an excellent evaluation metric for assessing the performance of object detection tasks, but it is too coarse to gauge the performance of robotic picking. In contrast, gripping success, which calculates performance based on α values, provides more specific and fine-grained insights into robotic picking performance, as it allows for predictions of gripping success based on factors such as the gripper and environmental complexity. Additionally, the performance of precision and recall can also be used to predict the efficiency of agricultural tasks.

Table 3 and Figure 8 shows the performance of peach fruits. The trend is quite different from the apple dataset. Although YOLOv8 showed the best performance in COCO AP, it lagged behind Swin Transformer in gripping success precision starting from α = 0.4, and in recall, it demonstrated a significantly lower performance, being completely outperformed by the other two models. This issue is also influenced by the differences in the composition of the apple and peach datasets. The average fruit size in the peach dataset is much larger, with fewer fruits, and they are distributed more coarsely. Therefore, even a small difference can have a significant impact.

### 3.4. Evaluation Using Expanded Metrics

**Figure 9:**
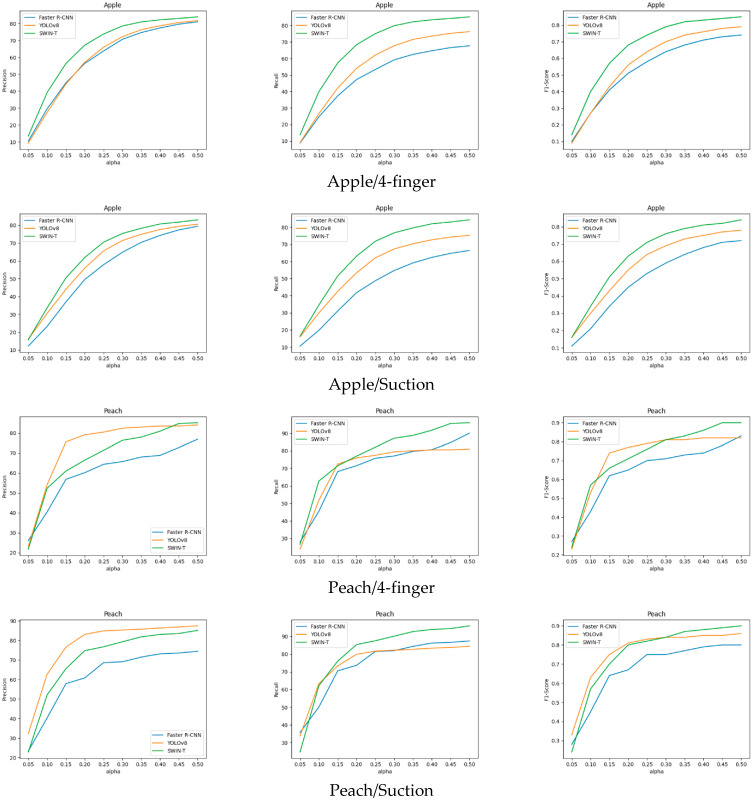
The gripping success results for the 4-finger type and suction-type grippers on the apple and peach datasets.

Figure 9 shows the experimental results of the expanded gripping success. The experiments were conducted using the apple and peach datasets, same as the previous version, and evaluations were performed for both four-finger type and suction type grippers. On the apple dataset, Swin Transformer achieved high success rates overall for both the four-finger and suction grippers. On the peach dataset, YOLOv8 had the highest precision, while for recall, Swin Transformer performed better overall with the four-finger gripper. For the suction gripper, YOLOv8 showed better performance when α≤0.1, while Swin Transformer performed better in the other range. The F1-score varied across different ranges, highlighting the need to select models based on the environment and the characteristics of the gripper being used. Additionally, for the same methodology, apples with smaller object sizes showed higher scores with the four-finger gripper compared to the suction gripper, while peaches with larger object sizes had higher scores with the suction gripper. This indicates that if images closer to the fruit can be captured, the suction-type gripper is more likely to be successful.

**Figure 10:**
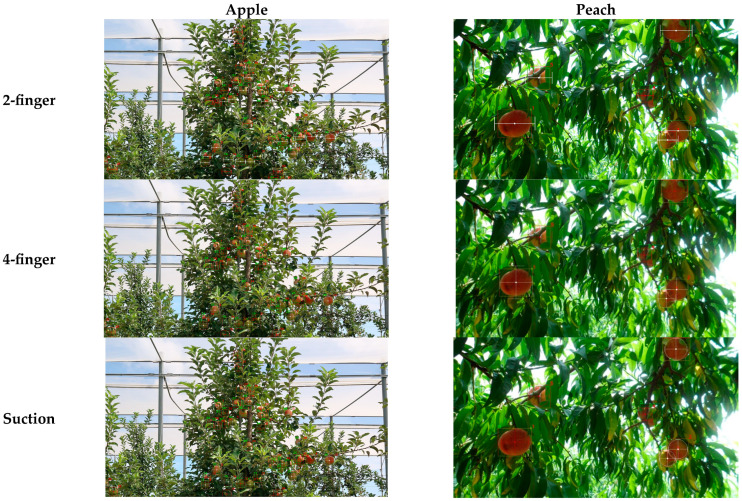
Comparisons of the gripping success for 2-finger, 4-finger, and suction-type grippers. The apple dataset was evaluated with Swin Transformer at α=0.1, and the peach dataset was evaluated with YOLO v8 at α=0.1. The numbers marked in green and red refer to the ground truth number and prediction number, respectively, and they may not match.

Figure 10 compares the gripping success evaluation results for the two-finger, four-finger, and suction-type grippers. The apple dataset was evaluated with Swin Transformer at α=0.1, while the peach dataset was evaluated with YOLOv8 at α=0.1. Since additional criteria were added for the four-finger gripper, grips that were successful with the two-finger gripper failed with the four-finger gripper.

## 4. Discussion

The gripping success metric is useful because the precision and recall can be interpreted with respect to the harvesting performance. The gripping success precision represents “how many fruits are successfully picked among the picking trials”, indicating the efficiency of the robotic task. The gripping success recall reflects “how many fruits are successfully picked out of total fruits in a tree”, which can be interpreted as the efficiency from the agricultural perspective. Users, such as farmers, can consider these two metrics to optimize the system for their specific needs. The F1-score, as the harmonic mean of precision and recall, provides an integrated measure and can be useful for comprehensive detection model selection.

**Figure 11:**
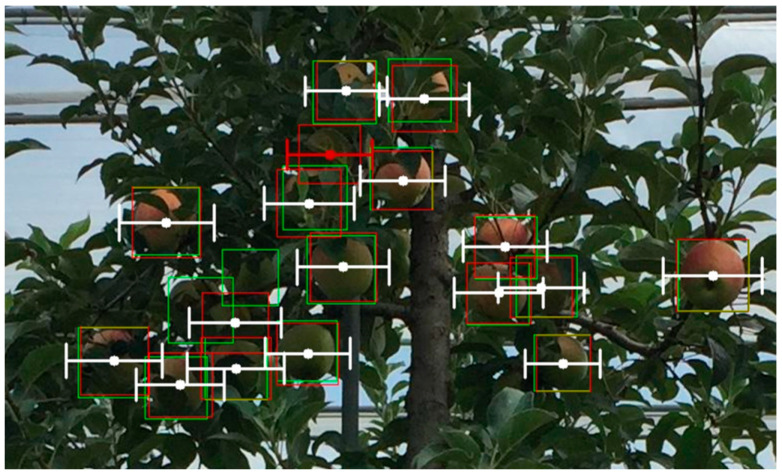
The evaluation output of YOLO v8 on the apple dataset with α=0.2. Successful grips are in white lines and failed grips are in red lines.

The proposed metric is based solely on two-dimensional images and considers only horizontal or vertical angles, representing a fundamental theoretical approach. It does not account for the gripper’s angle or any obstacles around the targets. In real-world robotic picking tasks, robots must consider three-dimensional spatial information and require six-DOF data for target objects. The example image in Figure 11 shows the evaluation output of YOLOv8 on the apple dataset with α=0.2. Multiple successful grips are generated, represented by white lines. However, the apples at the bottom-left and middle-right are hanging very densely, and both the grips and the GT bounding boxes are overlapping. Additionally, their depth positions vary. In such situations, some apples may not be pickable in reality, even if the grips are deemed successful in the two-dimensional space.

As such, success in a 2D simulation does not guarantee success in real-world applications. Future studies should focus on evaluating optimized grips, including angles, while incorporating more detailed spatial information such as depth and obstacles.

## 5. Conclusions

This paper addressed the limitations of conventional evaluation metrics for object detection, such as AP scores, in predicting the success of robotic picking. To overcome these issues, we proposed a novel evaluation metric, gripping success. The experimental results demonstrated that gripping success provides more detailed and appropriate insight for robotic applications compared to COCO AP.

The gripping success metric provides a more practical evaluation for robotic harvesting, particularly when object detection approaches are applied in agricultural automation. However, this study only considers two-dimensional picking scenarios, and the results may not fully translate to three-dimensional environments in the real world. In addition, the proposed metric does not account for gripper orientation or obstacles.

Future studies should incorporate three-dimensional spatial information, including depth data and obstacle consideration, to enhance the applicability of gripping success. The gripping success metric can serve as a foundation for advancing robotic harvesting technologies and contribute to the development of agricultural automation.

## Figures and Tables

**Table 1:** The detailed information of the used dataset.

	Apple [23]	Peach [21]
Variety	Hongro	Mihong
Number of images	199	125
Label type	Bounding box	Polygon mask
Total labels	13,260	1077
Average number of labels	67	9
Subsets for training	160:39	99:13:13

**Table 2:** Comparison of COCO AP and gripping success metrics for different object detectors on the apple dataset. The best performance for the metric is marked in bold.

Detector	COCO Metric			Gripping Success			
				α	Precision	Recall	F1-Score
**Faster R-CNN**	AP50 AP75		71.4 34.6	0.05	52.82	43.46	0.48
				0.10	68.27	56.66	0.62
				0.15	76.47	63.60	0.69
				0.20	80.57	67.16	0.73
				0.25	83.09	69.31	0.76
				0.30	84.83	70.78	0.77
				0.35	85.70	71.52	0.78
				0.40	86.09	71.84	0.78
				0.45	86.39	72.12	0.79
				0.50	86.47	72.20	0.79
	AP50-95	37.1			79.07	65.87	0.72
**YOLO v8**	AP50 AP75		84.1 49.9	0.05	41.53	38.67	0.40
				0.10	61.63	57.29	0.59
				0.15	72.17	67.18	0.70
				0.20	77.54	72.17	0.75
				0.25	80.60	75.10	0.78
				0.30	82.28	76.68	0.79
				0.35	83.06	77.43	0.80
				0.40	83.95	78.24	0.81
				0.45	84.07	78.36	0.81
				0.50	84.30	78.60	0.81
	AP50-95	48.7			75.11	69.9	0.72
**Swin** **Transformer**	AP50 AP75		**86.3** **56.7**	0.05	56.70	57.31	0.57
				0.10	72.65	73.69	0.73
				0.15	79.10	80.19	0.80
				0.20	82.89	84.03	0.83
				0.25	84.60	85.79	0.85
				0.30	85.38	86.58	0.86
				0.35	85.92	87.16	0.87
				0.40	86.06	87.30	0.87
				0.45	86.27	87.49	0.87
				0.50	86.31	87.53	0.87
	AP50-95	**52.3**			**80.59**	**81.71**	**0.81**

**Table 3:** Comparisons of evaluation metrics for a peach. The best performance for the metric is marked in bold.

Detector	COCO Metric		Gripping Success			
		AP	α	Precision	Recall	F1-Score
**Faster R-CNN**	AP50 AP75	86.5 65.2	0.05	55.06	65.35	0.60
			0.10	65.83	78.27	0.72
			0.15	69.51	82.27	0.75
			0.20	70.34	83.15	0.76
			0.25	72.90	85.72	0.79
			0.30	75.84	88.90	0.82
			0.35	76.29	89.30	0.82
			0.40	76.29	89.30	0.82
			0.45	76.29	89.30	0.82
			0.50	76.93	89.94	0.83
	AP50-95	57.2		71.53	84.15	0.77
**YOLO v8**	AP50 AP75	89.4 **71.5**	0.05	64.77	57.28	0.61
			0.10	77.29	73.68	0.75
			0.15	78.91	75.20	0.77
			0.20	82.12	78.96	0.81
			0.25	82.89	79.82	0.81
			0.30	82.89	79.82	0.81
			0.35	84.00	80.94	0.82
			0.40	84.00	80.94	0.82
			0.45	84.00	80.94	0.82
			0.50	84.00	80.94	0.82
	AP50-95	**64.9**		**80.49**	76.85	0.78
**Swin** **Transformer**	AP50 AP75	**90.0** 65.6	0.05	61.05	71.03	0.66
			0.10	73.32	83.90	0.78
			0.15	77.82	88.32	0.83
			0.20	78.30	88.83	0.83
			0.25	79.07	89.69	0.84
			0.30	80.68	91.45	0.86
			0.35	81.28	92.09	0.86
			0.40	85.12	95.94	0.90
			0.45	85.12	95.94	0.90
			0.50	85.99	96.75	0.91
	AP50-95	60.6		78.78	**89.39**	**0.84**

## Data Availability

Data is contained within the article. The original contributions presented in this study are included in the article.
